# Clinically proven markers of metastasis predict metastatic spread of human melanoma cells engrafted in scid mice

**DOI:** 10.1038/sj.bjc.6603594

**Published:** 2007-01-30

**Authors:** A Thies, S Mauer, O Fodstad, U Schumacher

**Affiliations:** 1 Zentrum für Experimentelle Medizin, Institut für Anatomie II: Experimentelle Morphologie, Universitätsklinikum Hamburg Eppendorf, Martinistrasse 52, Hamburg D-20246, Germany; 2 Department of Tumour Biology, The Norwegian Radium Hospital, Montebello, Oslo N-0310, Norway

**Keywords:** CEACAM1, HPA, L1, melanoma model, scid mouse

## Abstract

Metastasis formation is a complex process and as such can only be modelled *in vivo*. As markers indicating metastatic spread in syngenic mouse models differ significantly from those in man, this study aimed to develop a human melanoma xenograft mouse model that reflects the clinical situation. Six human melanoma cell lines (LOX, MV3, FEMX-1, G361, MeWo and UISO-Mel6) were xenografted into severe combined immunodeficient mice and tumour growth, metastatic behaviour and number of lung metastases were assessed. Tumours and metastases were analysed for HPA binding and expression of CEACAM-1 and L1, all markers indicative of metastasis in clinical studies. Development of primary tumour nodules ranged from 3 weeks (MV3) to 3 months (MeWo). Whereas G361 and FEMX-1 rarely formed lung metastases, MeWo, MV3 and LOX were moderately and UISO-Mel6 was highly metastatic. Similar to clinical studies, HPA, CEACAM1 and L1 indicated metastatic spread in the xenograft melanoma model, but were not all simultaneously expressed in all cell lines. Considering the strongest expression of one marker combined with an absent or low expression of the other two markers, we conclude that LOX is the cell line of choice for analyses of the functional role of HPA-binding glycoconjugates, UISO-Mel6 is ideally suited to study CEACAM1 function in melanoma spread and L1 function can best be modelled using MeWo.

Malignant melanoma, being the most aggressive form of skin cancer, represents a considerable clinical problem worldwide, where about 1 : 35 Caucasians will suffer from malignant melanoma during their lifetime with an incidence furthermore accelerating (Lens and Dawes, 2004). If the primary melanoma can be excised completely before it has spread to distant sites, the patient is cured. However, if metastases occur the patient's fate is sealed, as in spite of intensive research no curative treatment is yet available (Garbe and Blum, 2001; Eigentler *et al*, 2003). In order to make progress in the field of metastasis treatment, the process of malignant melanoma spread has to be better understood. Metastasis formation is a complex process, which involves many different steps, each of which has to be passed sequentially in order to lead to a clinically detectable metastasis (Hart and Saini, 1992). As this whole metastatic cascade is such a complex process, simple *in vitro* experiments can only partially mimick the course of metastatic spread and only animal experiments of metastasis can represent the full picture of this multistep phenomenon (Eccles, 2001).

In melanoma metastasis research, the mouse B16 melanoma model has found widespread application (Tao *et al*, 1982). However, as this model represents a syngenic mouse model, it suffers the inherent problems of all murine models of metastasis, namely whether it is directly transferable to humans. Concerning carbohydrate expression, which has profound influence on the metastatic behaviour of tumour cells (Schumacher *et al*, 2005), murine B16 melanoma cells significantly differ from human melanoma cells. Expression of WGA and PHA-L indicate metastatic spread of murine B16 melanoma cells in mice (Tao *et al*, 1982); however, both lectins were unable to predict metastatic spread in a clinical study (Thies *et al*, 2001). A further disadvantage of murine melanoma models is that murine melanocytes are exclusively located at the papilla of the hair, whereas human melanocytes are additionally found in the basal layer of the epidermis; thus, the biological situation is different. A further disadvantage of the syngenic mouse model is that all antibodies directed against human-specific antigens are not applicable.

To overcome these problems, human tumour cells xenograft animal model systems have been developed. In a number of cancers, particularly in breast and colon cancers, human cancer cell lines transplanted into severe combined immunodeficient (scid) mice have resulted in model systems, which resemble the clinical situation closely (Schumacher and Adam, 1997; Valentiner *et al*, 2005).

During tumour metastasis, modulation of cell adhesion molecule expression and aberrant glycosylation of the tumour cells play a key role in the metastatic cascade. Simultaneously, in clinical studies of malignant melanoma, binding of the lectin HPA indicating aberrant glycosylation, and upregulation of the cell adhesion molecules CEACAM1 and L1, have been correlated with metastatic spread (Thies *et al*, 2001, 2002a, 2002b).

The aim of the present study was to investigate the metastatic behaviour of human melanoma cells engrafted in scid mice. The metastatic pattern of these cell lines were compared with markers which have been useful to identify the metastatic phenotype of malignant melanomas in clinical studies.

## MATERIALS AND METHODS

### Cell lines

The human melanoma cell lines UISO-Mel6 (established from a primary malignant melanoma; see Rauth *et al* (1998)), MV3 (established from a metastatic melanoma lymph node; see Edward (2001)) and MeWo (established from a metastatic melanoma lymph node of a white, 78-year-old male; see Carey *et al* (1976)) were kindly provided by the Klinik für Dermatologie, Universitätsklinikum Hamburg-Eppendorf, Germany. The human melanoma cell lines LOX and FEMX-1 were both established from a metastatic lymph node (Fodstad *et al*, 1988a, 1988b). The human melanoma cell line G361 (established from a primary malignant melanoma from a 31-year-old Caucasian woman) was obtained from the American Type Culture Collection (ATCC No. CRL-1424). Cells were cultured under standard cell culture conditions (37°C, 100% relative humidity, 5% CO_2_) in RPMI medium (Gibco, Paisley, Scotland) supplemented with 10% heat-inactivated foetal bovine serum (FBS, Gibco), 2 mM L-glutamine (Gibco), 100 U ml^−1^ penicillin and 100 *μ*l ml^−1^ streptomycin (Gibco). For injection, the melanoma cells were harvested by trypsinisation, tested for viability and were adjusted to a concentration of 5 × 10^6^ viable cells per 1 ml cell culture. For glycoconjugate profiling of cell lines *in vitro*, cells of each cell line were fixed in 4% neutral-buffered formaldehyde and embedded in 4% agar before being processed to paraffin wax.

### Testing for mycoplasma infection

All cell lines were routinely tested for mycoplasma infection using the PCR-based VenorGeM Mycoplasma Detection kit (Minerva Biolabs GmbH, Berlin, Germany), following supplier's instructions. All cell lines were mycoplasma free.

### Animals

The methodology for carrying out the animal experiments was consistent with the UKCCR guidelines for the welfare of animals in experimental neoplasia (Workman *et al*, 1997). The experiment was recommended and supervised by the institutional animal welfare officer, and the local licensing authority (Behörde für Soziales, Familie, Gesundheit, Verbraucherschutz; Amt für Gesundheit und Verbraucherschutz; Billstr. 80, D-20539 Hamburg, Germany) approved the application for the experiment and assigned the project No. F1/8/01.

Pathogen-free male balb/c severe combined immunodeficient scid/scid mice aged 9–14 weeks were housed in filter-top cages and were provided with sterile water and food *ad libitum*. Mice weighed 20–25 g at the beginning of the experiment. All manipulations were carried out aseptically inside a laminar flow hood. Mice were randomly grouped, with each group consisting of 10 animals for each cell line. 10^6^ cells (in 200 *μ*l medium) were injected subcutaneously between the scapulae of each scid mice. The mice were killed when the tumour weight had reached 10% of the weight of the mice or when the tumours started to ulcerate. For duration of the experiment in each group, see Table 1a.

### Histology

Mice were killed by cervical dislocation, and the tumours were excised within their capsule, weighed and immediately fixed in 4% neutral-buffered formalin. The lungs of all the animals were dissected out and fixed in the same way for investigation of the presence and number of lung metastases. Lungs were cut under a dissecting microscope into 1-mm thick slices, which were spread randomly over a glass slide and then embedded in 4% warm liquefied agar. Slices were pressed down gently with a glass piston to avoid floating of the slices within the agar during the cool down process. The solidified agar blocks were then routinely processed for wax histology. Paraffin blocks of the lungs were serially sectioned. Numbers of lung metastases were analysed using the simplified quantitative method standardised in our laboratory as described by Jojovic and Schumacher (2000). Briefly, every 10th section of each lung was retained and 10 sections from the middle of the block were stained for H&E and the number of lung metastases was counted at a × 100 magnification. The mean value of numbers of lung metastases in the 10 sections for each lung (mean value_10_) was calculated. This mean value_10_ minus 20% was multiplied by the total number of serial sections of the respective lung in order to achieve an estimated value for the total number of lung metastases. Furthermore, the anatomical site of lung metastases (intravasal *vs* extravasal; pulmonary artery, bronchial vessels and intraseptal veins) was recorded.

### Lectin histochemistry

Paraffin sections (5 *μ*m) were processed for lectin- and immunohistochemistry using an avidin–biotin–alkaline phosphatase staining technique as has been reported previously (Thies *et al*, 2001, 2002a, 2002b).

Briefly, tissue sections were deparaffinised and rehydrated through a series of graded ethanols. After pretreatment with 0.1% trypsin (type II, crude, from porcine pancreas, Sigma, St Louis, MO, USA) in lectin buffer (0.05 M TRIS-buffered saline pH 7.6, with 1 mM CaCl_2_ and 1 mM MgCl_2_ added), slides were incubated sequentially with 10 *μ*g ml^−1^ biotinylated lectins (HPA, WGA, PHA-L; Sigma, St Louis, MO, USA) for 1 h at room temperature. Slides were then incubated with an avidin–alkaline phosphatase complex (Vectastain, Vector, ABC kit, Burlingame, CA, USA) for 30 min and enzyme reactivity of the alkaline phosphatase complex was visualised using Naphtol-AS-bisphosphate as a substrate and hexatozised New Fuchsin was used for simultaneous coupling. Slides were counterstained with Mayer's haemalum diluted 1:1 in distilled water for 10 s, blued under running tap water and mounted with Crystal Mount (Biomeda, Foster City, CA, USA).

For HPA, an additional, indirect staining method, following a standard protocol described earlier (Brooks *et al*, 1996) was performed. The only variation to the described method by Brooks *et al* (1996) was that the binding sites were visualised using an alkaline phosphatase complex instead of a peroxidase complex (Thies *et al*, 2001). Slides were pretreated as described above, but were then incubated with native unconjugated HPA (10 *μ*g ml^−1^) for 1 h at room temperature. After an incubation with 10% normal swine serum (DAKO, Glostrup, Denmark) for 30 min, slides were incubated overnight with a 1 : 100 diluted rabbit anti-HPA antibody (EY Lab., San Mato, CA, USA) at 4°C. Slides were then incubated with a 1 : 300 diluted biotinylated swine anti-rabbit antibody (DAKO, Glostrup, Denmark) for 30 min, followed by an incubation with a biotin–streptavidin–alkaline phosphatase complex (Vectastain, Vector, ABC kit, Burlingame, CA, USA). Developing enzyme reactivity, counterstaining and mounting was performed as described above.

Negative controls were treated the same way as preincubating the lectins with their respective nominal sugar. Serial sections of a primary human cutaneous melanoma, which proved to intensively bind the respective lectin in former studies (Thies *et al*, 2001) served as appropriate positive control and for evaluation of staining intensity.

### Immuno-histochemistry

Details on the production and characterisation of the monoclonal CEACAM1 antibody 4D1/C2 (in house preparation by Christoph Wagener (CW)) and the polyclonal L1 antibody (L1-S; in house preparation by Meliha Schachner (MS)) have been published previously (Haspel *et al*, 2000).

For antigen retrieval, slides were either microwaved at 500 W five times for 2 min in 10 mmol l^−1^ citrate buffer (pH 6.0), (CEACAM1), or exposed to 0.015 g protease (Sigma, Steinheim, Germany) dissolved in 150 ml TBS at 37°C for 7 min (L1). Nonspecific binding was blocked by 10% normal rabbit serum (CEACAM1) or 10% normal swine serum (L1). This was followed by an overnight incubation with 4D1/C2 antibody at 8 *μ*g ml^−1^, or by the 1 : 100 diluted polyclonal rabbit anti-human L1 antibody. Sections were then incubated with 1 : 40 diluted, biotinylated rabbit anti-mouse (CEACAM1) or biotinylated swine anti-rabbit (L1) antibody, followed by an incubation with an avidin–alkaline phosphatase complex (Vectastain, Vector, ABC kit, Burlingame, CA, USA) and antibody binding was visualised as described above.

Negative controls were treated the same way except replacing the primary antibody by the isotype-matched IgG (CEACAM1: Mouse IgG2a, κ UPC-10; Sigma; L1: Mouse IgG1; NatuTec, Frankfurt, Germany).

Central nervous myelin, as represented by an optic nerve, further served as the appropriate negative control for L1 staining. Cases of human primary malignant melanoma, which proved to express the CEACAM1 and L1 molecules in former studies (Thies *et al*, 2002a, 2002b) served as appropriate positive controls and as a standard for analysis of staining intensities. Additionally, for L1-positive control, a human peripheral nerve was used.

### Evaluation of staining pattern and statistical analyses

Two independent observers recorded the number of lung metastases and staining of the melanoma cells in the sections of primary tumours and lung metastases, which were encoded. Additionally, the intensity of positive staining from weak (+) to very intense staining (+++) was recorded. For documentation, the slides were examined under a Zeiss Axioplan photomicroscope and photographed digitally with a Zeiss MRc5 camera.

Correlation between the weight of the primary tumour and the number of respective lung metastases was analysed with a two-tailed Spearman rank correlation (Rko) using Graph Pad Prism 4.0 (Intuitive Software for Science, San Diego, CA, USA). *P*<0.05 was regarded as statistically significant.

## RESULTS

### Tumour growth in scid mice, metastatic potential and glycoconjugate expression

**Figure 1:**
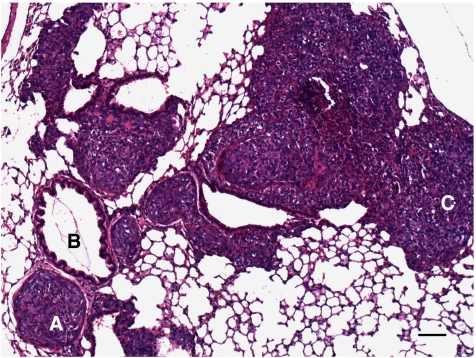
H&E staining of UISO-Mel6 lung metastasis. UISO-Mel6 developed lung metastases of enormous size (up to 900 cells in a cross-section). (**A**) Metastasis in the pulmonary artery; (**B**) bronchial branch; (**C**) extravasal melanoma cells. Bar represents 100 *μ*m.

All six mycoplasma-free cell lines engrafted in scid mice metastasised to the lungs; however, considerable variation concerning their growth in scid mice, their metastatic potential and glycoconjugate expression was observed (as summarised in Table 1; cell lines are listed according to their metastatic potential from low to high). Metastases of all six cell lines were located both intravasal in either the pulmonary artery, bronchial vessels, or the intraseptal veins and extravasal in the central or peripheral lung tissue (Figure 1). In general, glycoconjugate expression in primary tumour (PT) and corresponding spontaneous lung metastasis (LM) was almost always identical.

### G361

G361 cells developed primary tumours in all 10 scid mice, with an end point tumour growth of 40 days and a mean tumour weight of 0.93 g (range 0.4–1.4 g). Only 3/10 animals developed spontaneous lung metastases with a mean of only 75 metastases per lung (range 23–146). Primary tumours of six animals showed moderate (++) CEACAM1 immunoreactivity and weak (+) L1 immunoreactivity. G361 cells of PTs and LMs showed constant intense (+++) iHPA binding and weak (+) bHPA binding. In parallel to the other five cell lines, G361 cells were intensively labelled with WGA. No binding sites for PHA-L were observed in this cell line.

### FEMX-I

**Figure 2:**
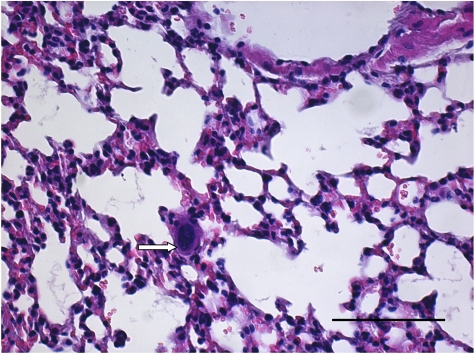
H&E staining of FEMX-1 lung metastasis. FEMX-1 developed only single-cell micrometastasis (arrow). Bar represents 100 *μ*m.

FEMX-I developed primary tumours in all 10 scid mice, with an end point tumour growth of 40 days and a mean tumour weight of 0.78 g (range 0.4–1.3 g). Seven out of 10 animals developed spontaneous lung metastases, with a mean of 226 metastases per lung (range 240–480). FEMX-1 developed only micrometastases consisting of one to five cells (Figure 2), which could not be evaluated immuno-histochemically. This is in contrast to all other cell lines, in which larger metastases had developed. Cells in the PTs of all 10 animals showed homogenous intense (+++) CEACAM1 immunoreactivity. L1 was weakly (+) expressed in the cells of 7/10 PTs. Cells in all PTs were homogenously labelled with iHPA (+) and bHPA (+ to ++) and WGA (+++). PHA-L bound to tumour cells in PTs of two animals.

### MeWo

**Figure 3:**
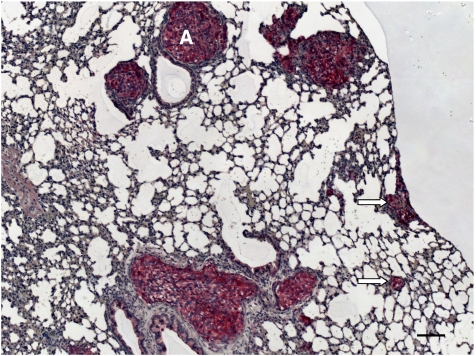
L1 immunohistochemistry of MeWo lung metastasis. Melanoma cells show intense (+++) L1 immunoreactivity, and even small metastases in the peripheral lung tissue are easy to detect (arrows). (**A**) metastasis in the pulmonary artery. Bar represents 100 *μ*m.

All 10 scid mice inoculated with MeWo cells developed primary tumours, with an end point tumour growth of 90 days and a mean tumour weight of 0.82 g (range 0.3–1.3 g). Six animals developed spontaneous lung metastases with a mean of 2860 metastases per lung (range 749–12 168). MeWo primary tumours and lung metastases showed equivocal intensity of glycoconjugate expression. Primary tumours and LMs were intensively (+++) L1-positive (Figure 3) and weakly (+) CAECAM1-positive. Lectin histochemistry showed homogenous moderate (++) bHPA binding. In contrast to the bHPA method, iHPA showed heterogenous weak (+) to intense (+++; PTs) or weak(+) to moderate (++) labelling of MeWo cells. WGA bound intensively (+++) to MeWo cells in PTs and LMs. No PHA-L-binding sites were observed.

### MV3

All 10 scid mice inoculated with MV3 cells developed primary tumours, with an end point tumour growth of 20 days and a mean tumour weight of 1.13 g (range 0.6–1.8 g). Eight animals developed spontaneous lung metastases with a mean of 196 metastases per lung (range 19–672). In general, MV3 PTs) and LMs showed homogenous glycoconjugate expression. Either all PTs and LMs expressed the respective CAMs or bound the lectins or no expression/binding was found. Staining intensities did not vary between PTs and LMs. Primary tumours and lung metastases expressed L1 (+), whereas CEACAM1 was not expressed in either PTs or LMs. Concerning lectin binding, PTs and LMs were weakly (+) labelled with iHPA but negative for bHPA. WGA-binding sites were intensively (+++) expressed in all PTs and LMs.

### LOX

**Figure 4:**
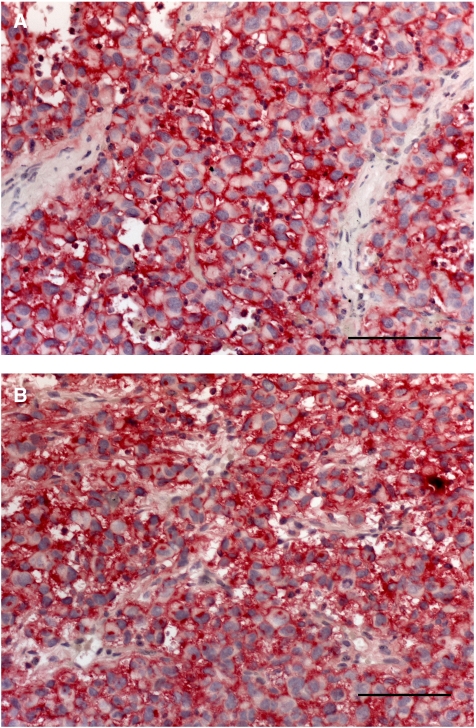
Comparative HPA lectinhistochemistry using two different methodological approaches. Primary tumours of the cell line LOX showed intense (+++) membranous HPA labelling with both methods: (**A**) bHPA using biotinylated HPA and (**B**) iHPA using native HPA and an anti-HPA antibody. Bar represents 100 *μ*m.

Two of 10 animals inoculated with LOX cells died of unknown reasons other than tumour growth. The remaining eight animals developed PTs with an end point tumour growth of 25 days and a mean tumour weight of 1.56 g (range 0.6–2.4 g). All eight animals developed spontaneous lung metastases with a mean of 168 metastases per lung (range 33–355). Glycoconjugate expression in PT and corresponding spontaneous lung metastasis was similar. LOX cells expressed L1 (+), but not did not express CEACAM1. Lectin histochmistry revealed intense (+++) HPA labelling of all cells in all PTs and LMs with both methods (Figure 4) and intense (+++) WGA labelling. No binding sites for PHA-L were observed.

### UISO-Mel6

**Figure 5:**
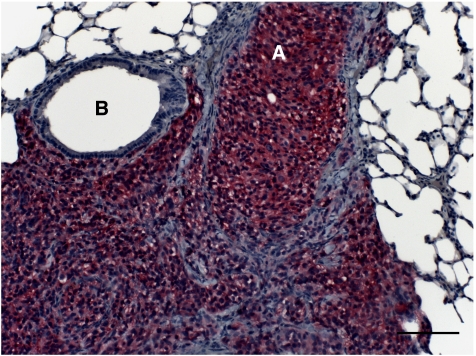
CEACAM1 immunohistochemistry of an UISO-Mel6 lung metastasis. The cells in the metastatic nodules exhibit intense (+++) CEACAM1 immunoreactivity. Melanoma cells have invaded the lung tissue starting from the (**A**) pulmonary artery. (**B**) Bronchial branch. Bar represents 100 *μ*m.

All 10 scid mice inoculated with UISO-Mel6 cells developed primary tumours, with an end point tumour growth of 90 days and a mean tumour weight of 0.89 g (range 0.1–1.5 g). All 10 animals developed spontaneous lung metastases of enormous size (up to 900 cells in a cross-section; Figure 1), with a mean of 24 031 metastases per lung (range 10 032–43 668). UISO-Mel6 cells exhibited moderate (++) to intense (+++) CEACAM1 expression in 8/10 PTs (two PTs were negative for CEACAM1) and intense (+++) CEACAM1 expression in the lung metastases of all 10 animals (Figure 5). L1 was expressed in the cells of all 10 PTs and in the cells of the lung metastases in all 10 animals. It was striking to note that the intensity of L1 immunoreactivity was significantly increased in the lung metastases (PTs: +; LMs: +++). Cells in 3/10 PTs and corresponding LMs were weakly (+) labelled with bHPA compared with the iHPA method, where cells in 5/10 PT and cells of all LMs were weakly (+) labelled. Cells in all PTs and LMs were intensively (+++) labelled with WGA. PHA-L-positive cells were recorded in the PT of one and in the LMs of two animals.

### Correlation between tumour weight and number of lung metastases

No correlation between weight of the primary tumour and numbers of spontaneous lung metastases existed for any of the six cell lines (Table 2), and no critical tumour weight for metastasis was apparent.

### Glycoconjugate expression and immunoreactivity *in vitro* compared with *in vivo*

Considerable differences between the glycoconjugate expression of paraffin-embedded *in vitro* cell lines and the paraffin-embedded *in vivo* tumours and metastases were obvious. Lectin histochemical and immunohistochemical results of paraffin-embedded cell lines *in vitro* are summarised in Table 3. All six cell lines expressed L1, which is in parallel to the *in vivo* results, however, to considerably different extents. CEACAM1 was only expressed by FEMX-1 (+++) and G361 (++), contrasting *in vi*vo data, where UISO-Mel6 (+++) and MeWo (+) also showed CEACAM1 expression. HPA binding *in vitro* was comparable with that *in vivo*.

## DISCUSSION

This study aimed at developing a clinically relevant melanoma model. For this purpose, tumour growth and metastatic behaviour of six different human melanoma cell lines subcutaneously xenografted into scid mice was analysed and correlated with the expression of proven markers of metastasis in clinical studies (Thies *et al*, 2001, 2002a, 2002b, 2006). The scid mouse was employed as a host in the xenograft system as it has already proved useful as a clinically relevant xenograft host for the metastatic spread of human breast and colon cancer cell lines (Schumacher and Adam, 1997). Furthermore, subcutaneous injection of tumour cells, as presented here, warrants that the metastatic cascade as a whole can be analysed, whereas intravenous (iv) injection of tumour cells, as sometimes described by others, results in dissemination rather than in metastasis (Kjonniksen *et al*, 1994). For malignant melanoma, in particular, subcutaneous injection has the advantage of being an almost orthotopic model and therefore, reflects the clinical situation from the anatomical point of view more closely than the aforementioned iv injection. Considerable differences between the glycoconjugate expression of *in vitro* grown cells and the *in vivo* tumours resulting from the growth of injected cells and their metastases in our study further underlines the considerable importance of whole *in vivo* model systems for the study of metastasis.

All cells from all six cell lines engrafted in scid mice, but as expected, the time frame for the development of primary tumours varied considerably between the cell lines, ranging from 3 weeks (MV3) to 3 months (UISO-Mel6, MeWo). Somewhat surprisingly, cells from all cell lines formed spontaneous metastases in the lungs. However, no correlation between the metastatic rate and the number of lung metastases was found, as has been described for HT29 colon cancer cell lines and MDA MB 435 breast cancer cell lines transplanted into scid mice (Schumacher and Adam 1997; Valentiner *et al*, 2005). Furthermore, no threshold tumour weight existed for any cell line, again contrasting with our results in colon and breast cancer cell lines. However, this observation correlates with the clinical situation of melanoma spread, as there are patients presenting with thin melanomas at the time of surgery who suffer from early metastasis and death whereas other patients with thick melanomas survive (Balch *et al*, 2000).

UISO-Mel6 cells produced spontaneous lung metastases in all animals and formed extremely high numbers of lung metastases, and thus can be regarded as highly metastatic. Despite a 100% metastatic rate, LOX cells formed only moderate numbers of lung metastases in the mice, and MeWo cells produced high numbers of lung metastases but metastasised in only 60% of the animals. MV3 cells formed moderate numbers of lung metastases and had a metastatic rate of 80%. Hence, LOX, MeWo and MV are intermediately metastatic. Although, FEMX-1 showed a metastatic rate of 70%, it formed only few lung metastases and G361 rarely metastasised to the lungs (metastatic rate 30%) and also developed few numbers of metastases. These two cell lines can thus be regarded as poorly metastatic.

FEMX-1, however, is so far particular with its metastatic pattern, as FEMX-1 cells formed only micrometastases, consisting of one to five cells, which therefore, could not be evaluated histochemically. Primary tumours of FEMX-1 cells reached a size and weight comparable to those of the other cell lines within a time frame of 90 days; therefore, micrometastases are not due to low tumourigenicity or short time-period. FEMX-1 xenografts might thus present a model system to analyse melanoma dormancy, the conceptual framework to explain a prolonged quiescence state (beyond 10 years), in which tumour cells are present, but tumour progression is not clinically apparent, as has been described in approximately one-third of the melanoma patients (De Giorgi *et al*, 2003). To further analyse this hypothesis, metastatic behaviour of FEMX-1 cells in mice after surgical excision of the primary tumour should be analysed, which we are onto in our laboratory.

Correlating the metastatic behaviour of the six different human melanoma cell lines in scid mice and their respective glycoconjugate expression, those markers indicating metastatic spread in clinical studies (Thies *et al*, 2001, 2002a, 2002b) were also indicative of metastases in our xenograft model, and alike the clinical situation (Thies *et al*, 2006), marker expression was conserved in the distant metastases.

The highly metastatic cell line UISO-Mel6 broadly confirmed that CEACAM1 is a marker of metastasis in the human melanoma scid mouse model as well. Primary tumours and LMs of all animals inoculated with UISO-Mel6 cells showed intense CEACAM1 expression and only low expression of the other markers. MeWo and G361 PTs and LMs also expressed CEACAM1, however, to a lower extent, and showed strong additional marker expression. Therefore, UISO-Mel6 is the cell line of choice for analyses of CEACAM1 function in melanoma metastasis.

In parallel to our clinical results (Thies *et al*, 2002b), all six cell lines expressed L1 in the cells of all PTs and LMs, with MeWo showing the most intense L1 expression. Strikingly, intensity of L1 immunoreactivity was even upregulated in UISO-Mel6 lung metastases, which again reflects the clinical situation (Thies *et al*, 2006) and supports the hypothesis of a functional role of L1 in melanoma metastasis. As a result of its intense L1 expression in all PTs and LMs and its low expression of the other markers, the cell line MeWo is ideally suited to further analyse L1 function in melanoma metastasis.

HPA-binding glycoconjugates, having a well-established role in cancer research (Schumacher *et al*, 2005), were expressed in five of six tumorigenic and metastatic cell lines. In parallel to our clinical investigation (Thies *et al*, 2002a), we compared two different methodological approaches for HPA binding analyses, demonstrating that in the animal model both methods are warranted. LOX is the cell line of choice for study of the functional role of HPA-binding carbohydrates in melanoma metastasis, as it shows intense HPA binding, does not express CEACAM1 and shows only low L1 expression. LOX further shows good tumour engraftment, has a sizeable tumour grown within 25 days and a 100% metastasis rate. Also, intensely HPA-positive and metastatic cell line MeWo shows a tumour growth phase of 90 days, which has to be taken into consideration, concerning therapeutic studies.

Kjonniksen *et al* (1994) demonstrated that the metastatic cell line LOX showed strong HPA binding, which is in parallel to our results. Additional results by that group showed, that the HPA-negative cell line FEMX-1 was not metastatic after iv injection, which is in contrast to our results, where all primary FEMX-1 tumours expressed HPA-binding sites and developed metastatic deposits in the lungs of 7/10 mice. However, FEMX-1 metastases frequently consisted of only one to five cells contrasting metastases of the other cell lines. A simple explanation may, therefore, be that these metastatic cells have been overlooked. A possible further explanation is given by microbial contamination in this cell line. We have established routine screening for mycoplasma, as about 30% of the permanent cell lines transferred to our facilities are mycoplasma infected. Earlier xenograft experiments with FEMX-1 and MeWo (data not shown) showed that both cell lines were indeed less tumorigenic, did not metastasise into the lungs and were HPA-negative, comparable to the results by Kjonniksen *et al* (1994). They further did not express CEACAM1 (MeWo) and/or L1 (MeWo, FEMX-1). Subsequent tests for mycoplasma infection demonstrated broad contamination of both cell lines with mycoplasma. Our results presented here, using only mycoplasma free cell lines, reversed these results, in part, and demonstrate the considerable influence of mycoplasma contamination on the carbohydrate expression, tumorigenic and metastatic potential of tumour cells, as has also been reported by others (Uphoff and Drexler, 2004). Therefore, stringent controls for and prevention of mycoplasma contamination should be standard and should be sought before any cell experiment proof of a mycoplasma free-cell culture.

We furthermore analysed binding of the lectins PHA-L and WGA, which indicated metastatic spread of murine B16 melanoma cells (Tao *et al*, 1982), but are not correlated with melanoma metastasis in man (Thies *et al*, 2001). In accordance to clinical results, our human melanoma cell line xenograft model showed no significance of PHA-L or WGA-binding glycoconjugates in melanoma metastasis, and its clinical relevance is therefore superior to that of the B16 melanoma model.

In parallel to clinical studies (Thies *et al*, 2006), not all markers of metastatic spread were simultaneously expressed in all metastatic cell lines, indicating the complexity of the metastatic cascade and the heterogeneity of the tumour cells. The inherent advantage of the model presented here is that because some cell lines express all markers (UISO-Mel6; highest metastatic potential, MeWO, G361) and others express only one or two markers, the functional role of the respective carbohydrates can be analysed alone and in combination with each other. CEACAM1, L1 and HPA-binding carbohydrates (GalNAc/GlucNAc) might have multiple function supporting melanoma metastasis, namely invasion into and migration through the ECM as well as adhesion to the endothelium and transendothelial migration or tumour vascularisation (Ergün *et al*, 2000; Voura *et al*, 2001; Gutwein *et al*, 2003; Ebrahimnejad *et al*, 2004; Schumacher *et al*, 2005), which can now be analysed in our model system.

Besides their clinical relevance, these models own the advantage that all human-specific antibodies can be used for preclinical therapeutic studies, which are based upon recognition of human-specific sequences.

Concluding, the human melanoma xenograft scid mouse model presented in this study (i) closely reflects the clinical situation and (ii) underlines the complexity of metastasis formation. It is therefore, ideally suited to investigate the functional role of CAMs and carbohydrates both separately and in combination with each other.

## Figures and Tables

**Table 1:** (a) Characteristics of six human melanoma cell lines xenografted into scid mice (*n*=10 per cell line) and (b) glycoconjugate expression of their respective primary tumours and spontaneous lung metastasis

**Cell line**	**Origin**		**Duration (days)**		**Mean tumour weight**			**Metastasis rate (%)**		**Mean number of lung metastases**		
(*a*)												
G361	PT		40		0.93			30		75		
FEMX-1	LNM		40		0.78			70^a^		226		
MeWo	LNM		90		0.69			60		2860		
MV3	LNM		20		1.13			80		196		
LOX	LNM		25		1.56			100		168		
UISO-Mel6	PT		90		0.89			100		24 031		
												

**Cell line**	**L1**		**CEACAM1**		**bHPA**		**IHPA**		**WGA**		**PHA-L**	
	**PT**	**LM**	**PT**	**LM**	**PT**	**LM**	**PT**	**LM**	**PT**	**LM**	**PT**	**LM**
(*b*)												
G361	+	+	++	++	+	+	+++	+++	+++	+++	−	−
FEMX-1	+	∅	+++	∅	+/++	∅	+	∅	+++	∅	−	∅
MeWo	+++	+++	+	+	++	++	+/++/+++	++/+++	+++	+++	−	−
MV3	+	+	−	−	−	−	+	+	+++	+++	−	−
LOX	+	+	−	−	+++	+++	+++	+++	+++	+++	−	−
UISO-Mel6	+	+++	++/+++	+++	+	+	+	+	+++	+++	−	−

Cell lines are listed according to their metastatic potential from low to high.

a FEMX-1 developed only micrometastases, consisting of one to five cells. LNM=lymph node metastasis; PT=primary tumour.

Staining intensity was recorded as (–) negative, (+) weak, (++) moderate and (+++) intense. ∅: FEMX-1 developed only single-cell micrometastases, which could not be evaluated immunohistochemically. PT=primary tumour; LM=lung metastasis.

**Table 2:** Correlation of tumour weight and number of lung metastasis of human melanoma cell lines subcutaneously xenografted into scid mice

**Cell line**	**Spearman *r***	**95% CI**	***P***
G361	−0.18	−0.75–0.55	0.65
FEMX-1	0.40	−0.12–0.75	0.12
MeWo	0.61	−0.03–0.89	0.06
LOX	0.04	−0.69–0.72	0.94
MV3	0.30	−0.40–0.78	0.39
UISO-Mel6	−0.16	−0.74–0.57	0.69

The number of lung metastases was not correlated with the weight of the primary tumour in any of the six cell lines.

CI=confidence interval.

**Table 3:** Comparative analysis of glycoconjugate expression of six human melanoma cell lines *in vitro* (a) and *in vivo* (b)

**Cell line**	**L1**	**CEACAM-1**	**bHPA**	**iHPA**	**WGA**	**PHA-L**
(*a*) *In vitro*						
G361	+++	++	+	+	+++	+/++
FemX-I	+++	+++	++	+	+++	++
MeWo	++	−	+	++	+++	+
MV3	+	−	−	+	+++	+++
LOX	++	−	+++	++	+++	++
UISO-Mel6	++	−	+	++	+++	−/+/++
						
(*b*) *In vivo*						
G361	+	++	+	+++	+++	−
FemX-I	+	+++	+/++	+	+++	−
MeWo	+++	+	++	+/++/+++	+++	−
MV3	+	−	−	+	+++	−
LOX	+	−	+++	+++	+++	−
UISO-Mel6	+	++/+++	+	+	+++	−

Expression of markers *in vitro* differed considerably from that of *in vivo*.

## References

[bib1] Balch CM, Buzaid AC, Atkins MB, Cascinelli N, Coit DG, Fleming ID, Houghton AJ, Kirkwood JM, Mihm MF, Morton DL, Reintgen D, Ross MI, Sober A, Soong SJ, Thompson JA, Thompson JF, Gershenwald JE, McMasters KM (2000) A new American Joint Committee on Cancer staging system for cutaneous melanoma. Cancer 88: 1484–1491 10717634 10.1002/(sici)1097-0142(20000315)88:6<1484::aid-cncr29>3.0.co;2-d

[bib2] Brooks SA, Lymboura M, Schumacher U, Leathem AJ (1996) Histochemistry to detect Helix pomatia lectin binding in breast cancer: methodology makes a difference. J Histochem Cytochem 44: 519–524 8627008 10.1177/44.5.8627008

[bib3] Carey TE, Takahasi T, Rsnick LA, Oettgen HF, Old LJ (1976) Cell surface antigens of human malignant melanoma; mixed hemadsorption assays for humoral immunity to cultured autologous melanoma cells. Proc Natl Acad Sci USA 73: 3278–3282 1067619 10.1073/pnas.73.9.3278 PMC431008

[bib4] De Giorgi V, Massi D, Germini G, Mannone F, Quercioli E, Carli P (2003) Immediate local recurrence after the excision of a polypoid melanoma: tumor dormancy or tumor activation? Dermatol Surg 29: 664–667 12786716 10.1046/j.1524-4725.2003.29163.x

[bib5] Ebrahimnejad A, Streichert T, Nollau P, Horst AK, Wagener C, Bamberger AM, Brummer J (2004) CEACAM1 enhances invasion and migration of melanocytic and melanoma cells. Am J Pathol 165: 1781–1787 15509546 10.1016/S0002-9440(10)63433-5 PMC1618678

[bib6] Eccles SA (2001) Basic principles for the study of metastasis using animal models. In Metastasis Research Protocols, Volume II, Analysis of Cell Behaviour *In Vitro* and *In Vivo*, Brooks SA and Schumacher U (eds), pp 161–171. Humana Press: Totowa, New Jersey 10.1385/1-59259-137-X:161 21340856

[bib7] Edward M (2001) Melanoma cell-derived factors stimulate glycosaminoglycan synthesis by fibroblast cultured as monolayers and within contracted collagen lattices. Br J Dermatol 144: 465–470 11260000 10.1046/j.1365-2133.2001.04069.x

[bib8] Eigentler TK, Caroli UM, Radny P, Garbe C (2003) Palliative therapy of disseminated malignant melanoma: a systematic review of 41 randomised clinical trials. Lancet Oncol 4: 748–759 14662431 10.1016/s1470-2045(03)01280-4

[bib9] Ergün S, Kilik N, Ziegeler G, Hansen A, Nollau P, Gotze J, Wurmbach JH, Horst A, Weil F, Fernando M, Wagener C (2000) CEA-related cell adhesion molecule 1: a potent angiogenic factor and major effector of vascular endothelial growth factor. Mol Cell 5: 311–320 10882072 10.1016/s1097-2765(00)80426-8

[bib10] Fodstad O, Aamdal S, McMenamin M, Nesland JM, Pihl A (1988a) A new experimental metastasis model in athymic nude mice, the human malignant melanoma LOX. Int J Cancer 75: 442–449 10.1002/ijc.2910410322 3346110

[bib11] Fodstad O, Kjonniksen I, Aamdal S, Nesland JM, Boyd MR, Pihl A (1988b) Extrapulmonary tissue-specific metastasis formation in nude mice injected with FEMX-I human melanoma cells. Cancer Res 48: 4382–4388 3390834

[bib12] Garbe C, Blum A (2001) Epidemiology of cutaneous melanoma in Germany and worldwide. Skin Pharmacol Appl Skin Physiol 14: 280–290 11586069 10.1159/000056358

[bib13] Gutwein P, Mechtersheimer S, Riedle S, Stoeck A, Gast D, Joumaa S, Zentgraf H, Fogel M, Altevogt DP (2003) ADAM10-mediated cleavage of L1 adhesion molecule at the cell surface and in released membrane vesicles. FASEB J 17: 292–294 12475894 10.1096/fj.02-0430fje

[bib14] Hart IR, Saini A (1992) Biology of tumour metastasis. Lancet 339: 1453–1461 1376386 10.1016/0140-6736(92)92039-i

[bib15] Haspel J, Friedlander DR, Ivgy-May N (2000) Critical and optimal Ig domains for promotion of neurite outgrowth by L1/Ng-CAM. J Neurobiol 42: 287–302 10645969

[bib16] Jojovic M, Schumacher U (2000) Quantitative assessment of spontaneous lung metastases of human HT29 colon cancer cells transplanted into SCID mice. Cancer Lett 152: 151–156 10773406 10.1016/s0304-3835(99)00443-7

[bib17] Kjonniksen I, Rye PD, Fodstat O (1994) Helix pomatia agglutinin in human tumour cell lines: correlation with pulmonary metastases in nude mice. Br J Cancer 69: 1021–1024 8198963 10.1038/bjc.1994.200 PMC1969443

[bib18] Lens MB, Dawes M (2004) Global perspectives of contemporary epidemiological trends of cutaneous malignant melanoma. Br J Dermatol 150: 179–185 14996086 10.1111/j.1365-2133.2004.05708.x

[bib19] Rauth S, Green A, Kichima J, Shilkaitis A (1998) Supression of tumorigenic and metastatic potentials of human melanoma cell lines by mutated (143Val-Ala) p53. Br J Cancer 77: 2215–2222 9649136 10.1038/bjc.1998.369 PMC2150410

[bib20] Schumacher U, Adam E (1997) Lectin histochemical HPA-binding pattern of human breast and colon cancers is associated with metastasis formation in severe combined immunodeficient mice. Histochem J 29: 677–684 9413741 10.1023/a:1026404832394

[bib21] Schumacher U, Brooks SA, Mester J (2005) The lectin Helix pomatia agglutinin as a marker of metastases-clinical and experimental studies. Anticancer Res 25: 1829–1830 16033108

[bib22] Tao TW, Burger MM, Finne J, Prieels JP (1982) The influence of membrane mutations on metastasis. Biosci Rep 2: 597–599 7139074 10.1007/BF01314221

[bib23] Thies A, Berlin A, Brunner G, Schulze H-J, Moll I, Pfüller U, Wagener C, Schachner M, Altevogt P, Schumacher U (2006) Glycoconjugate profiling of primary melanoma and its sentinel node and distant metastases: implications for diagnosis and pathophysiology of metastases. Cancer Lett [E-pub ahead of print 3 July 2006] 10.1016/j.canlet.2006.05.020 16822608

[bib24] Thies A, Moll I, Berger J, Schumacher U (2001) Lectin binding to cutaneous malignant melanoma: HPA is associated with metastasis formation. Br J Cancer 84: 819–823 11259098 10.1054/bjoc.2000.1673 PMC2363810

[bib25] Thies A, Moll I, Berger J, Wagener C, Brümmer J, Schulze H-J, Brunner G, Schumacher U (2002a) CEACAM1 Expression in cutaneous malignant melanoma predicts the development of metastatic disease. J Clin Oncol 20: 2530–2536 12011132 10.1200/JCO.2002.05.033

[bib26] Thies A, Schachner M, Moll I, Berger J, Schulze H-J, Brunner G, Schumacher U (2002b) Overexpression of the cell adhesion molecule L1 is associated with metastasis in cutaneous malignant melanoma. Eur J Cancer 38: 1708–1716 12175686 10.1016/s0959-8049(02)00105-3

[bib27] Uphoff CC, Drexler G (2004) Detecting mycoplasma contamination in cell cultures by polymerase chain reaction. Methods Mol Med 88: 319–326 14634244 10.1385/1-59259-406-9:319

[bib28] Valentiner U, Hall DBS, Brooks SA, Schumacher U (2005) HPA binding and metastasis formation of human breast cancer cell lines transplanted into severe combined immunodeficient (scid) mice. Cancer Lett 219: 233–242 15723724 10.1016/j.canlet.2004.07.046

[bib29] Voura EB, Ramjeesingh RA, Montgomery AM, Siu CH (2001) Involvement of integrin alpha(v)beta(3) and cell adhesion molecule L1 in transendothelial migration of melanoma cells. Mol Biol Cell 12: 2699–2710 11553709 10.1091/mbc.12.9.2699 PMC59705

[bib30] Workman P, Twentyman P, Balkwill F, Balmain A, Chaplin D, Double J, Embleton J, Newell D, Raymond R, Stables J, Stephens T, Wallace J (1997) United Kingdom Co-ordinating Committee on Cancer Research (UKCCCR) guidelines for the welfare of animals in experimental neoplasia (second edition). Br J Cancer 77: 1–10 10.1038/bjc.1998.1 PMC2151254 9459138

